# Early Recurrence Following Complete Initial Resection Predicts Adverse Oncological Outcomes in NMIBC

**DOI:** 10.3390/jcm15062463

**Published:** 2026-03-23

**Authors:** Yavuz Mert Aydın, Necmettin Aydın Mungan

**Affiliations:** Department of Urology, Zonguldak Bülent Ecevit University, Kozlu, 67639 Zonguldak, Türkiye; naydin.mungan@beun.edu.tr

**Keywords:** non-muscle-invasive bladder cancer (NMIBC), early recurrence, transurethral resection of bladder tumor (TUR-BT), prognostic factors, oncological outcomes

## Abstract

**Background/Objectives:** Early recurrence after complete initial transurethral resection of bladder tumor (TUR-BT) may indicate biologically aggressive non-muscle-invasive bladder cancer (NMIBC). This study aimed to identify clinicopathological predictors of ER and its independent impact on progression and survival outcomes. **Methods:** Clinical data of 335 primary NMIBC patients who underwent TUR-BT between 2012 and 2024 were retrospectively analyzed. Patients with non-primary tumors, incomplete resection, or follow-up <6 months were excluded from the study. Patients were categorized into recurrence-free, early recurrence, and late recurrence groups. Logistic regression was used to identify predictors of early recurrence. Progression-free survival (PFS), cancer-specific survival (CSS), and overall survival (OS) were analyzed using the Kaplan–Meier method and Cox regression. A 36-month landmark analysis was conducted to adjust for heterogeneity in follow-up duration. **Results:** Early recurrence occurred in 118 patients (35.2%). Independent predictors of early recurrence were tumor size (OR = 1.012, *p* = 0.038), T1 stage (OR = 2.57, *p* = 0.004), high-grade pathology (OR = 1.933, *p* = 0.030), and absence of single-dose intravesical chemotherapy (IVC) (OR = 3.642, *p* = 0.025). Additionally, adjuvant IVC (OR = 0.279, *p* = 0.015) and intravesical BCG (OR = 0.427, *p* = 0.006) independently reduced the risk of early recurrence. Early recurrence independently predicted worse PFS (HR = 6.053), CSS (HR = 2.052), and OS (HR = 1.961) (all *p* < 0.001). The landmark analysis confirmed these results (all *p* < 0.05). **Conclusions:** Early recurrence after initial and complete TUR-BT is an independent predictor of adverse oncological outcomes. Identifying high-risk patients and applying early intravesical therapy may improve outcomes by preventing early recurrence.

## 1. Introduction

In 2022, bladder cancer accounted for 613,791 newly reported cases, making it the 9th most common cancer worldwide, and with 220,349 deaths, it ranked as the 13th leading cause of cancer-related mortality globally. The incidence rate is approximately 4 times higher in men (28.7 vs. 7.0 per 105), with the highest rates reported in Southern and Northern European countries. Apart from regional incidence variations, tobacco use, occupational exposures (e.g., aromatic amines), and arsenic in drinking water are key contributors to the etiopathogenesis of bladder cancer [1].

Approximately 75% of bladder cancers are diagnosed as non-muscle invasive bladder cancer (NMIBC) [2]. Depending on the clinicopathological characteristics, reported annual recurrence rates range from 15% to 61%, while progression rates range from 1% to 17% [3]. The standard diagnostic method and initial management of NMIBC is transurethral resection of the bladder tumor (TUR-BT), followed by risk-adapted intravesical therapy. Current guidelines recommend intravesical instillations of either chemotherapy or Bacillus Calmette–Guérin (BCG), depending on the patient’s risk category [2,4]. Despite these treatment modalities, NMIBC patients still show a heterogeneous prognosis and have insufficient oncological outcomes [5].

The foremost and most challenging issue in NMIBC management is the early identification of patients at risk of progression and the implementation of aggressive treatment strategies. To address this, the European Organization for Research and Treatment of Cancer (EORTC) and the Club Urologico Español de Tratamiento Oncológico (CUETO) study groups developed nomograms, which were subsequently revised over time to encompass patients receiving intravesical BCG instillations. EORTC and CUETO studies have consistently demonstrated that recurrence is primarily associated with tumor burden (e.g., multifocality, prior recurrences, concomitant CIS) and female sex. In contrast, progression is driven mainly by tumor biology (stage, grade) and, importantly, by early recurrence [3,5,6,7]. Despite findings indicating the prognostic significance of early recurrence, the factors associated with early recurrence have not been thoroughly examined in the literature. Furthermore, the prognostic differences between early and late recurrence in NMIBC have been rarely and inconsistently evaluated. Therefore, the present study aims to address a significant gap in the literature by investigating predictors of early recurrence and comparing oncological outcomes between those with early and late recurrence.

## 2. Materials and Methods

In this study, the records of 488 patients who underwent TUR-BT at our center between January 2012 and October 2024 were reviewed. Exclusion criteria included patients who had previously undergone TUR-BT at another center with a diagnosis of bladder tumor, those with incomplete resection, those in whom muscle-invasive bladder cancer was identified on initial TUR-BT, those with less than 6 months of follow-up, and patients with incomplete medical records or missing key clinical data. After applying the exclusion criteria, all eligible patients were included in the study.

After ethics committee approval, clinical, pathological, and follow-up data of patients diagnosed with NMIBC were retrospectively collected from institutional records. Baseline demographic variables included age, sex, and American Society of Anesthesiologists (ASA) score. Tumor characteristics recorded included the number of lesions (and whether they were unifocal or multifocal), tumor size, morphology (papillary, solid, or flat), pathological stage (Ta, T1, T2, carcinoma in situ [CIS]), and tumor grade. Surgical details comprised completeness of initial transurethral resection and perioperative intravesical chemotherapy (IVC) instillation. Treatment-related variables included intravesical therapy regimens such as IVC and BCG induction and maintenance. Oncological outcomes assessed were recurrence, progression, recurrence-free survival (RFS), progression-free survival (PFS), cancer-specific survival (CSS), and overall survival (OS). Follow-up information included cystoscopic recurrence and progression, progression to invasive Transitional Cell Carcinoma (TCC), and the cause of death (bladder cancer–related or other causes). Recurrence was defined as the pathological confirmation of malignancy following resection of a tumor detected during follow-up cystoscopy. Progression was defined more broadly than the conventional criterion of muscle-invasive disease (T2 or higher) [5,6]. In the present study, progression was defined as any evidence of worsening of the disease compared with the initial tumor, including stage upstaging (e.g., Ta to T1), grade progression (e.g., low-grade to high-grade), an increase in the number of tumors, or an increase in tumor size. Early recurrence was defined as the detection of a pathologically confirmed tumor at the first surveillance cystoscopy, whereas late recurrence was defined as the presence of a pathologically confirmed tumor during surveillance in patients who were tumor-free at their first surveillance cystoscopy. The timing of early recurrence was typically around the third month; however, cystoscopies performed earlier or up to approximately 4.5 months for any reason were also considered as the first surveillance cystoscopy.

In our institution, follow-up of NMIBC patients was performed in accordance with international guideline recommendations (American Urological Association [AUA] and European Association of Urology [EAU]) [2,4]. Surveillance consisted of cystoscopy and urine cytology at 3-month intervals during the first 2 years, every 6 months between years 3 and 5, and annually thereafter. High-risk patients were monitored more intensively, whereas low-risk patients followed a less stringent protocol. Intravesical therapy was administered according to guideline-based risk stratification. Patients in the low-risk group received a single immediate postoperative instillation of IVC only. Patients in the intermediate-risk group received IVC or BCG instillation for one year. Patients in the high- and very high-risk groups received full-dose intravesical BCG (induction followed by a 3-year maintenance therapy). A single-dose immediate IVC refers to an instillation administered within 24 h of TUR-BT. For IVC, patients were considered to have received treatment if they completed at least 6 weeks of induction therapy [8]. BCG instillations were performed according to the SWOG protocol [9]. In the high- and particularly the very high-risk group, early cystectomy was considered an alternative treatment option; however, these patients were excluded from the study. Patients were considered to have received intravesical BCG therapy if they met the FDA’s definition of adequate dosing, which requires at least 5 of 6 doses in the initial induction course and at least 2 of 3 doses in maintenance therapy [10].

The study analyzed factors predicting early recurrence and investigated independent variables associated with PFS, CSS, and OS. In addition, a sensitivity analysis was performed using the conventional definition of progression (≥T2) to assess whether the association between early recurrence and PFS remained consistent. The cohort was subsequently divided into three groups: Group 1 comprised recurrence-free patients; Group 2 included patients with early recurrence; and Group 3 included patients with late recurrence. PFS, CSS, and OS were compared across the groups. For survival analyses, patients who had not experienced the event by the last follow-up or were lost to follow-up were censored at the last contact date. Furthermore, a 36-month landmark analysis was conducted to account for the wide interquartile range (IQR) of follow-up durations. After excluding the patients who had not reached 36-month follow-up, Kaplan–Meier analysis and the log-rank test were recalculated for PFS, CSS, and OS.

### 2.1. Ethics Statement

The Clinical Research Ethics Committee of The Zonguldak Bulent Ecevit University approved the study protocol (approval number: 2025/18-12). The study protocol adhered to the principles outlined in the Declaration of Helsinki.

### 2.2. Statistical Analysis

The dataset was screened for missing values prior to statistical analysis. No missing data were identified for the variables included in the analyses; therefore, all patients were included in the statistical models. Categorical variables were compared using the chi-square test and Fisher’s exact test. Normality analysis of continuous data was evaluated using the Shapiro–Wilk and Kolmogorov–Smirnov Tests. Independent T-test and ANOVA were used when comparing normally distributed variables, while Mann–Whitney and Kruskal–Wallis tests were used for non-normally distributed variables. ANOVA was performed for post hoc analysis, and Tamhane’s test was applied when the assumption of equal variances was not met. For non-normally distributed continuous variables, the Kruskal–Wallis test was used for overall group comparisons, followed by Dunn’s post hoc test with Bonferroni correction for pairwise comparisons. Logistic regression was employed to analyze factors predicting early recurrence, whereas Cox regression was used for the analysis of PFS, CSS, and OS. Multicollinearity was assessed using variance inflation factors (VIF), and model performance was evaluated using the Hosmer–Lemeshow test and receiver operating characteristic (ROC) analysis. Variables included in the multivariable models were selected based on clinical relevance and statistical significance in univariate analyses. Survival analysis was performed using the Kaplan–Meier method and the log-rank test. *p* < 0.05 was considered to indicate a statistically significant result. SPSS software IBM SPSS Statistics for Windows, version 25.0 (IBM Corp., Armonk, NY, USA) was used for the analyses.

## 3. Results

A total of 335 patients were included in the study, consisting of 119 recurrence-free patients (Group 1), 118 patients with early recurrence (Group 2), and 98 patients with late recurrence (Group 3). The median age of the entire cohort was 69.9 (IQR:15.4) years, the majority of patients were male (*n*: 289, 86.3%), and the median follow-up was 44.5 (IQR: 64.1) months.

### 3.1. Clinicopathological Characteristics

Patients in Group 1 were significantly younger (Group 1: 66.7 [IQR 15.3] years vs. Group 2: 70.8 [IQR 15.9] years, *p* < 0.001; Group 1 vs. Group 3: 70.3 [IQR 16.5] years, *p* = 0.001) and had fewer tumor number (Group 1: 1 [IQR 1] vs. Group 2: 2 [IQR 2], *p* = 0.001; Group 1 vs. Group 3: 1 [IQR 2], *p* = 0.029). Patients in Group 2 had larger tumor sizes (Group 1: 30 [IQR 20] mm vs. Group 2: 50 [IQR 40] mm, *p* < 0.001; Group 2 vs. Group 3: 35 [IQR 40] mm, *p* = 0.001), and their PFS was significantly shorter than that of the other two groups (Group 1: 66.1 [IQR 66.6] months vs. Group 2: 5.8 [IQR 25.3] months, *p* < 0.001; Group 2 vs. Group 3: 38.6 [IQR 50.5] months, *p* < 0.001). Similarly, patients in Group 3 had larger tumor sizes (Group 1 vs. Group 3, *p* = 0.030) and shorter PFS compared with those in Group 1 (Group 1 vs. Group 3, *p* = 0.001). Patients in Group 2 had a significantly shorter OS compared with those in Groups 1 and 3 (Group 1: 66.1 [IQR 66.6] months, vs. Group 2: 26.9 [IQR 37.9] months, *p* < 0.001; Group 2 vs. Group 3: 54.0 [IQR 74.9], *p* < 0.001), while no significant difference in OS was observed between Groups 1 and 3 (Group 1 vs. Group 3 *p* = 0.341).

There were no statistically significant differences between the groups regarding gender or the presence of m. propria in first TUR-BT. While more than half of the patients in Group 1 (51.3%) were classified as ASA 1 and 2, a greater proportion of patients in Group 2 (62.7%) and Group 3 (59.2%) had ASA scores of 3 and 4 (*p* < 0.001). Papillary morphology was more frequent in Group 1 (84.0%) and Group 3 (74.5%), whereas the solid morphology rate (43.2%) was higher in Group 2 compared to the other groups (*p* < 0.001). The rate of T1 tumors was significantly higher in Group 2 (*p* < 0.001). The distribution of EAU risk categories also differed significantly (*p* < 0.001), with the “very high risk” category being more common in the early recurrence group. Single-dose IVC administration was significantly less frequent in the early recurrence group (3.4%) compared with the non-recurrent (31.1%) and late recurrence (17.3%) groups (*p* < 0.001). Similarly, receipt of intravesical therapy (either chemotherapy or BCG) was markedly different among the groups (*p* < 0.001). In Group 2, progression, bladder cancer mortality, all-cause mortality, and progression to invasive TCC were significantly higher than in the other groups (all *p*-values < 0.001) (Table 1).

### 3.2. Predictors of Early Recurrence

Univariable logistic regression analysis revealed that age, ASA score, tumor multiplicity, tumor size, tumor morphology, tumor pathology, tumor grade, absence of single-dose IVC, and intravesical treatment status were significantly associated with early recurrence.

In multivariable analysis, tumor size (OR = 1.012, 95% CI = 1.001–1.023, *p* = 0.038), T1 stage (OR = 2.57, 95% CI = 1.344–4.916, *p* = 0.004), high-grade pathology (OR = 1.933, 95% CI = 1.065–3.511, *p* = 0.030), absence of single-dose IVC (OR = 3.642, 95% CI = 1.178–11.266, *p* = 0.025) remained independent predictors of early recurrence. Additionally, IVC (OR = 0.279, 95% CI = 0.100–0.777, *p* = 0.015) and intravesical BCG (OR = 0.427, 95% CI = 0.233–0.785, *p* = 0.006) independently reduced the risk of early recurrence (Table 2).

The logistic regression model demonstrated good calibration (Hosmer–Lemeshow test, *p* = 0.901) and good discriminative ability (AUC = 0.818, 95% CI: 0.772–0.865). No evidence of multicollinearity was observed (VIF range: 1.09–1.68).

### 3.3. Progression-Free Survival (PFS)

During follow-up, 113 patients (33.7%) experienced progression. In univariable analysis, age, ASA3-4, tumor size, early recurrence, tumor morphology, tumor pathology, tumor grade, single dose IVC, and intravesical treatment were associated with PFS. In multivariable analysis, early recurrence was strongly associated with worse PFS (HR = 6.053, 95% CI = 3.806–9.628, *p* < 0.001), and T1 disease (HR = 1.795, 95% CI = 1.032–3.124, *p* = 0.038) was another independent predictor of worse PFS. Moreover, intravesical BCG therapy (HR = 0.532, 95% CI = 0.332–0.854, *p* = 0.009) was independently associated with improved PFS (Table 3).

**Figure 1:**
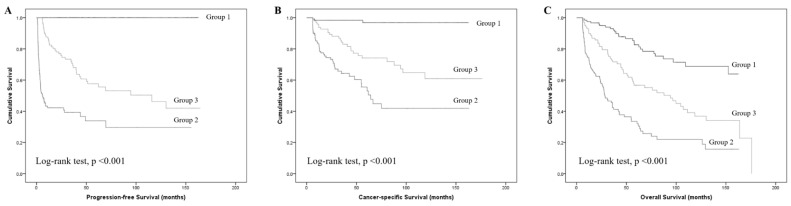
Kaplan–Meier curves for survival outcomes according to recurrence groups. (**A**) Progression-free survival (PFS); (**B**) cancer-specific survival (CSS); (**C**) overall survival (OS). Log-rank test, *p* < 0.001 for all comparisons.

Kaplan–Meier analysis demonstrated significantly shorter PFS in Group 2 compared to Groups 1 and 3 (log-rank *p* < 0.001). Figure 1A. In addition, a sensitivity analysis using the conventional ≥T2 progression definition yielded similar results, confirming that early recurrence remained an independent predictor of worse progression-free survival (Supplementary Table S1).

### 3.4. Cancer-Specific Survival (CSS)

In total, 74 patients (22.1%) died due to bladder cancer. Multivariable Cox regression revealed that early recurrence (HR = 2.052, 95% CI = 1.196–3.520, *p* = 0.009), solid morphology (HR = 2.058, 95% CI = 1.239–3.419, *p* = 0.005), and T1 stage (HR = 3.143, 95% CI = 1.425–6.934, *p* = 0.005) were independent predictors of poorer CSS.

Kaplan–Meier analysis demonstrated significantly shorter CSS in Group 2 compared to other groups (log-rank *p* < 0.001). Supplementary Table S2 and Figure 1B.

### 3.5. Overall Survival (OS)

Univariable analysis revealed that advanced age, high ASA score, increased tumor multiplicity, larger tumor size, early recurrence, solid morphology, T1 stage, high-grade pathology, absence of single-dose IVC, and lack of intravesical therapy were all associated with poor overall survival. Multivariable analysis identified advanced age (HR = 1.031, 95% CI = 1.012–1.051, *p* = 0.001), ASA score (*p* = 0.008), early recurrence (HR = 1.961, 95% CI = 1.351–2.848, *p* < 0.001), solid morphology (HR = 1.664, 95% CI = 1.151–2.406, *p* = 0.007), and T1 stage (HR = 1.679, 95% CI = 1.069–2.636, *p* = 0.024) as independent predictors of reduced OS. Although individual ASA categories did not reach statistical significance, a clear trend toward decreased survival with increasing ASA class was observed (Table 4).

The Kaplan–Meier survival curve (Figure 1C) showed significantly poorer OS in patients with early recurrence compared to those with late recurrence or recurrence-free (log-rank *p* < 0.001).

Median OS was achieved in both the early and late recurrence groups, whereas it was not reached in the recurrence-free group.

After excluding patients who did not complete 36 months of follow-up for the landmark analysis, the recalculated log-rank tests based on 200 patients (Group 1: 94; Group 2: 42; Group 3: 64) remained statistically significant across all survival endpoints (PFS, CSS, and OS; *p* < 0.001; *p* < 0.001; *p* = 0.001, respectively).

## 4. Discussion

This study demonstrated that early recurrence following initial TUR-BT was significantly associated with poorer PFS, CSS, and OS. Even after landmark analysis, the prognostic differences in PFS, CSS, and OS persisted. This finding reinforces that the adverse prognostic impact of early recurrence is not an artifact of follow-up duration or censoring bias. Increased tumor size, T1 stage, and high-grade pathology were identified as independent predictors of early recurrence, whereas both single-dose IVC and adjuvant intravesical therapy (either chemotherapy or BCG) were associated with a reduced risk of early recurrence. Early recurrence and T1 pathology were independent risk factors for all oncological outcomes: PFS, CSS, and OS. Adjuvant intravesical BCG was associated with improved PFS but did not affect CSS or OS. Solid morphology was identified as an independent risk factor for both CSS and OS. Advanced age and a high ASA score independently predicted poorer OS but had no significant effect on CSS.

Several studies have examined factors predicting recurrence and progression in patients with NMIBC. In 2006, Sylvester et al. published a combined analysis of EORTC studies, in which they identified the previous recurrence rate, number of tumors, tumor size, T stage, concomitant CIS, and tumor grade as independent predictors of time to first recurrence and progression, and they developed a nomogram [3]. However, the recurrence and progression rates were overestimated because patients who received intravesical BCG were not included in this study. In contrast, the CUETO group included only patients treated with BCG in their studies and proposed a new scoring model. In this model, the calculated recurrence rates and progression rates among high-risk patients were lower than those in the EORTC model, reflecting the effect of BCG treatment. In these studies, factors associated with increased recurrence included female sex, recurrent tumor, tumor multiplicity, and concomitant CIS, whereas factors associated with a higher risk of progression included recurrent tumor, high-grade tumor, T1 stage, and tumor detected at the first cystoscopy [6,7]. Thus, the clinical significance of early recurrence was emphasized. In the CUETO group, the BCG maintenance duration was considerably shorter than in current practice; therefore, the EORTC group repeated their studies in patients who received BCG for 1–3 years [2,5]. In this study, early recurrence was independently associated with higher prior recurrence rate, multiple tumors, and high tumor grade; late recurrence was predicted by the prior recurrence rate and tumor multiplicity. Disease progression and CSS were significantly influenced by tumor stage (T1) and grade (high), while OS was primarily affected by patient age and tumor grade [5]. In routine clinical practice, not all NMIBC patients receive BCG therapy, and even among those who do, not everyone is able to complete the full treatment course. Consequently, in 2021, the EORTC group broadened the scope of their research and renewed their study using a cohort more representative of real-world clinical conditions [11].

In contrast to previous EORTC and CUETO studies, which included patients with potential residual disease and recurrent cases, our study focused exclusively on patients with newly diagnosed NMIBC and who underwent complete TUR-BT, thereby minimizing the confounding effects of residual tumor and prior treatments (intravesical therapy or TUR-BT). Moreover, in the EORTC and CUETO studies, early recurrence was not included in the regression analyses; thus, none of these studies evaluated early recurrence as an independent factor for oncological outcomes (PFS, CSS, and OS). These methodological considerations yield a more homogeneous and well-defined cohort, enabling a clearer evaluation of the prognostic significance of early recurrence and a more accurate assessment of the biological behavior of de novo NMIBC, thereby extending and refining the existing evidence. Furthermore, this study suggests that early recurrence may partly reflect the tumor’s underlying biological behavior rather than being solely attributable to surgical or therapeutic factors. Several molecular pathways have been implicated in bladder cancer pathogenesis, including alterations in FGFR3, PI3K/AKT, and MAPK signaling, which may promote tumor cell proliferation, survival, and disease progression [12]. Crucially, large-scale genomic analyses in NMIBC, such as the UROMOL study, have demonstrated that high-grade and T1 tumors are characterized by a high somatic mutation burden (e.g., TP53 and ERBB2) and frequent APOBEC-signature mutagenesis [13,14]. This reflects substantial genomic instability that may contribute to tumor heterogeneity and aggressive behavior. Consistent with these molecular observations, tumor size, T1 stage, and high-grade pathology were identified as independent predictors of early recurrence in our cohort. These findings suggest that the increased genomic instability reported in high-grade and T1 tumors may partly explain the higher likelihood of early recurrence and its association with adverse oncological outcomes.

Consistent with our findings, Jeong et al. reported that early recurrence adversely affected cystectomy-free survival and OS, compared with late recurrence and recurrence-free patients [15]. However, unlike our study, their analysis included only Ta patients and defined early recurrence using a 1-year threshold. When considering only Ta tumors, defining early recurrence as occurring within 1 year may be reasonable. However, when T1 cases are also included, this timeframe may not fully capture early recurrence. In our study, early recurrence was defined as recurrence detected during the first cystoscopy, typically performed at the third month. This approach aligns with the EORTC study’s methodology and may more accurately reflect the tumor’s aggressive biological behavior [5]. Brouzes et al. analyzed recurrences in two categories: any-grade and high-grade. Predictors for any-grade recurrence included a prior history of bladder cancer and tumor multiplicity, whereas predictors for high-grade recurrence were T1 stage, tumor multifocality, prior history of bladder tumor, and BCG interruption. In the same study, female gender, higher tumor stage, and a prior history of bladder cancer were identified as factors associated with disease progression [16]. Similarly, recent studies have suggested that refined pathological grading systems may further improve prognostic stratification in NMIBC. For instance, Ferro et al. demonstrated that hybrid grading systems combining the WHO 1973 and WHO 2004/2022 classifications may enhance the prediction of recurrence and progression in patients with Ta NMIBC [17]. Beyond tumor-specific markers, systemic inflammatory indices such as the systemic inflammation response index (SIRI) are emerging as valuable prognostic tools. Recent evidence suggests that SIRI can enhance risk stratification and provide complementary prognostic information, particularly in patients undergoing radical cystectomy [18]. In our study, the definition of progression encompassed both high-grade recurrences and progression, as described by Brouzes et al. Consistent with their findings, we identified the T1 stage and the absence of BCG therapy as independent predictors of progression.

We highlight that early recurrence was consistently associated with worse oncological outcomes, further supporting its prognostic relevance in NMIBC management. Teoh et al. suggest that early recurrence is predominantly related to local and technical factors, such as incomplete resection, undetected tumor remnants, or tumor reimplantation during TURBT, whereas late recurrence is more often due to field cancerization and de novo tumor formation [19]. In this context, the primary step to prevent early recurrence is to improve the quality of the initial TUR-BT. To minimize tumor re-implantation, either early single-dose IVC or continuous saline irrigation is recommended [2,4]. However, the evidence regarding the relative superiority of these two approaches remains conflicting [20]. Furthermore, our study found that adjuvant intravesical therapy (chemotherapy and BCG) was associated with a reduced risk of early recurrence. Although IVC can reduce early recurrence, it does not have an independent impact on progression or survival. In our study, the adjuvant therapy found to be effective in preventing progression was intravesical BCG. Nevertheless, consistent with the literature, we did not observe a significant effect of BCG therapy on CSS or OS [21,22].

To better distinguish the biological contribution of early recurrence from potential confounders such as residual disease, surgical quality, and intravesical therapy, we applied strict exclusion criteria and incorporated relevant variables into our multivariable models. Patients with incomplete resections were excluded to minimize the impact of residual disease. In addition, variables reflecting surgical quality and treatment factors, including the presence of detrusor muscle and the use of intravesical therapy, were included in the analysis. Despite these adjustments, early recurrence remained independently associated with PFS, CSS, and OS. These findings suggest that early recurrence may, at least in part, reflect underlying tumor aggressiveness; however, the potential influence of non-biological factors cannot be entirely excluded. Moreover, it is essential to interpret these findings with a balanced perspective regarding residual disease. While we strictly excluded patients with clinically documented incomplete resections, ‘complete resection’ is based on the surgeon’s macroscopic assessment. Consequently, microscopic tumor foci or very small lesions that may escape intraoperative detection could still remain and potentially contribute to early recurrence. Therefore, the possibility that a subset of early recurrences may reflect undetected residual tumor rather than purely biological tumor aggressiveness cannot be entirely excluded. In this context, identifying patients at risk of early recurrence (increased tumor size, T1 stage, and high-grade pathology) in advance and managing them with more aggressive therapeutic strategies may improve oncological outcomes.

Our study underscores the importance of immediate single-dose IVC and adjuvant intravesical therapy following TUR-BT. These interventions have the potential to improve oncological outcomes by reducing early recurrence and mitigating disease progression. Currently, the most widely used adjuvant intravesical therapies are single-dose IVC and BCG instillation. However, in patients at high risk of early recurrence, emerging agents such as intravesical gemcitabine, sequential gemcitabine–docetaxel, or novel immune- and gene-based therapies may offer promising alternatives and warrant further investigation in prospective trials.

Despite our efforts to adjust for known confounders, residual confounding cannot be excluded. Variability in surgical technique and resection thoroughness may have affected TURBT quality, as completeness was based on the surgeon’s macroscopic assessment rather than objective criteria. Although re-TURBT was performed according to guideline-based indications, variability in its timing and compliance may have introduced heterogeneity in progression outcomes. Differences in pathological assessment—including grading consistency and reporting of lamina propria invasion—may also affect staging accuracy across the study period. Furthermore, despite applying standard definitions of adequate intravesical BCG therapy, real-world variability in treatment adherence and scheduling cannot be fully excluded. Nevertheless, the consistent independent association of early recurrence with PFS, CSS, and OS across both primary and landmark analyses suggests that it may, at least in part, reflect underlying tumor biology beyond these clinical variabilities.

The International Bladder Cancer Group (IBCG) argued that relying solely on progression to muscle-invasive disease (≥T2) neglects clinically meaningful changes and proposed that progression should also encompass stage upstaging (Ta → T1) or grade increase in non-muscle-invasive tumors [23]. By adopting a broader definition of progression that includes grade progression, upstaging, and increases in tumor size and number, our study aligns with these perspectives. However, this approach has certain limitations that should be acknowledged. The broader definition of progression used in this study, which extends beyond the conventional ≥T2 criterion, may have led to an increased number of progression events. While this approach may better capture clinically relevant disease worsening, it may also limit direct comparability with studies using the conventional definition. In addition, including only patients who were initially diagnosed and underwent complete TUR-BT in this study minimized the potential confounding effects of residual disease and treatment heterogeneity. However, several limitations must be acknowledged. The retrospective design and single-center setting may introduce selection bias and limit the external validity of the findings. In addition, the risk-adapted treatment allocation inherent to real-world clinical practice may also introduce potential confounding factors. Although we employed rigorous inclusion criteria and multivariable analyses, unmeasured confounding factors such as surgeon experience or compliance with maintenance therapy might have influenced outcomes. Additionally, molecular or genomic tumor profiling, which could provide deeper insights into the biological mechanisms driving early recurrence, was not available.

## 5. Conclusions

Early recurrence following initial TUR-BT was independently associated with adverse oncological outcomes (shorter PFS, CSS, and OS). Tumor size, T1 stage, and high-grade pathology were identified as predictors of early recurrence, whereas single-dose immediate IVC and adjuvant intravesical BCG were associated with a reduced risk of early recurrence. Our findings suggest that early recurrence may not solely reflect surgical technical factors or residual disease, but may also be partially related to the tumor’s intrinsic biological behavior. Further prospective studies are needed to determine whether the timely identification of early recurrence patients and the adoption of more aggressive treatment strategies may improve oncological outcomes.

## Figures and Tables

**Table 1:** The clinicopathological characteristics of the patients.

		Group 1 (*n* = 119)	Group 2 (*n* = 118)	Group 3 (*n* = 98)	*p*-Value
**Age** (years)		66.7 (15.3)	70.8 (15.9)	70.3 (16.5)	**<0.001 ^a^**
**Tumor Multiplicity** (number)		1 (1)	2 (2)	1 (2)	**<0.001 ^b^**
**Tumor Size** (mm)		30 (20)	50 (40)	35 (40)	**<0.001 ^c^**
**Recurrence-Free Survival**		66.1 (66.6)	1.8 (1.6)	15.3 (25.8)	**<0.001 ^d^**
**Progression-Free Survival**		66.1 (66.6)	5.8 (25.3)	38.6 (50.5)	**<0.001 ^e^**
**Overall Survival**		66.1 (66.6)	26.9 (37.9)	54.0 (74.9)	**<0.001 ^f^**
**Age**	<70	73 (61.3%)	51 (43.2%)	48 (49.0%)	0.017 *
	≥70	46 (38.7%)	67 (56.8%)	50 (51.0%)	
**Gender**	Male	101 (84.9%)	101 (85.6%)	87 (88.8%)	0.684 *
	Female	18 (15.1%)	17 (14.4%)	11 (11.2%)	
**ASA**	1	14 (11.8%)	1 (0.8%)	1 (1%)	<0.001 *
	2	47 (39.5%)	43 (36.4%)	39 (39.8%)	
	3	54 (45.4%)	62 (52.5%)	52 (53.1%)	
	4	4 (3.4%)	12 (10.2%)	6 (6.1%)	
**Presence of M. propria**	No	46 (38.7%)	53 (44.9%)	41 (41.8%)	0.620 *
	Yes	73 (61.3%)	65 (55.1%)	57 (58.2%)	
**Tumor Multiplicity**	Unifocal	76 (63.9%)	51 (43.2%)	50 (51.0%)	0.006 *
	Multifocal	43 (36.1%)	67 (56.8%)	48 (49.0%)	
**Tumor Morphology**	Papillary	100 (84.0%)	65 (55.1%)	73 (74.5%)	<0.001 **
	Solid	17 (14.3%)	51 (43.2%)	25 (25.5%)	
	Flat	2 (1.7%)	2 (1.7%)	0 (0.0%)	
**Tumor Size**	<3 cm	54 (45.4%)	11 (9.3%)	37 (37.8%)	<0.001 *
	≥3 cm	65 (54.6%)	107 (90.7%)	61 (62.2%)	
**Tumor Pathology**	Ta	73 (61.3%)	23 (19.5%)	48 (49.0%)	<0.001 **
	T1	41 (34.5%)	91 (77.1%)	49 (50.0%)	
	Concomitant CIS	5 (4.2%)	4 (3.4%)	1 (1.0%)	
**EAU RISK**	Low	57 (47.9%)	5 (4.2%)	23 (23.5%)	<0.001 *
	Intermediate	21 (17.6%)	16 (13.6%)	21 (21.4%)	
	High	35 (29.4%)	72 (61.0%)	43 (43.9%)	
	Very High	6 (5.0%)	25 (21.2%)	11 (11.2%)	
**Single Dose IVC**	No	82 (68.9%)	114 (96.6%)	81 (82.7%)	<0.001 *
	Yes	37 (31.1%)	4 (3.4%)	17 (17.3%)	
**Intravesical** **Treatment**	No	46 (38.7%)	83 (70.3%)	49 (50.0%)	<0.001 *
	Chemotherapy	32 (26.9%)	6 (5.1%)	28 (28.6%)	
	BCG	41 (34.5%)	29 (24.6%)	21 (21.4%)	
**Progression**	No	119 (100%)	46 (39.0%)	57 (58.2%)	<0.001 *
	Yes	0 (0.0%)	72 (61.0%)	41 (41.8%)	
**Bladder Cancer Ex**	No	116 (97.5%)	73 (61.9%)	72 (73.5%)	<0.001 *
	Yes	3 (2.5%)	45 (38.1%)	26 (26.5%)	
**All-cause mortality**	No	93 (78.2%)	37 (31.4%)	45 (45.9%)	<0.001 *
	Yes	26 (21.8%)	81 (68.6%)	53 (54.1%)	
**Progression to** **Invasive TCC**	No progress	119 (100%)	74 (62.7%)	73 (74.3%)	<0.001 *
	Progress	0 (0.0%)	44 (37.3%)	25 (25.7%)	

^a^: Post hoc pairwise comparisons. **Group 1 vs. Group 2 *p* < 0.001, Group 1 vs. Group 3 *p* = 0.001**, and Group 2 vs. Group 3 *p* = 0.953. ^b^: Post hoc pairwise comparisons. **Group 1 vs. Group 2 *p* = 0.001, Group 1 vs. Group 3 *p* = 0.029, and** Group 2 vs. Group 3 *p* = 0.586. ^c^: Post hoc pairwise comparisons. **Group 1 vs. Group 2 *p* < 0.001**, **Group 1 vs. Group 3 *p* = 0.030**, **Group 2 vs. Group 3 *p* = 0.001.** ^d^: Post hoc pairwise comparisons. **Group 1 vs. Group 2 *p* < 0.001, Group 1 vs. Group 3 *p* < 0.001, Group 2 vs. Group 3 *p* < 0.001.** ^e^: Post hoc pairwise comparisons. **Group 1 vs. Group 2 *p* < 0.001, Group 1 vs. Group 3 *p* = 0.001, Group 2 vs. Group 3 *p* < 0.001.** ^f^: Post hoc pairwise comparisons. **Group 1 vs. Group 2 *p* < 0.001**, Group 1 vs. Group 3 *p* = 0.341, **Group 2 vs. Group 3 *p* < 0.001.** * Pearson Chi-Square Test Used, ** Fisher’s Exact Test Used. The median and interquartile range were reported for continuous variables. ASA: American Society of Anesthesiologists; M. Propria: Muscularis propria; CIS: carcinoma in situ; EAU: European Association of Urology; IVC: intravesical chemotherapy; BCG: or Bacillus Calmette–Guérin; TCC: Transitional Cell Carcinoma.

**Table 2:** Univariable and multivariable logistic regression analysis for predictors of early recurrence.

Factor		Univariable Analysis				Multivariable Analysis			
		OR	*p*-Value	%95 CI		OR	*p*-Value	%95 CI	
				Lower	Upper			Lower	Upper
**Age**	*Years*	1.031	**0.005**	1.009	1.053	1.013	0.349	0.986	1.042
**Gender**	*Male (R)* vs. *female*	1.091	0.791	0.572	2.081				
**ASA**	1		**0.049**				0.184		
	2	7.500	0.055	0.959	58.673	3.298	0.292	0.358	30.415
	3	8.774	**0.038**	1.131	68.040	2.789	0.373	0.293	26.597
	4	18.00	**0.010**	2.012	161.044	8.095	0.095	0.696	94.192
**Presence of M. Propria**	*Yes (R)* vs. *no*	1.218	0.393	0.774	1.917				
**Tumor M** **ultiplicity**	*Number*	1.244	**0.004**	1.073	1.444	1.096	0.322	0.914	1.315
**Tumor Size**	*Mm*	1.026	**<0.001**	1.017	1.035	1.012	**0.038**	1.001	1.023
**Tumor Morphology**	*Papillary (R)*		**<0.001**				0.374		
	*Solid*	3.232	**<0.001**	1.964	5.318	1.452	0.213	0.807	2.610
	*Flat*	2.662	0.333	0.367	19.288	2.537	0.442	0.237	27.194
**Tumor Pathology**	*Ta (R)*		**<0.001**				**0.017**		
	*T1*	5.319	**<0.001**	3.123	9.061	2.570	**0.004**	1.344	4.916
	*Concomitant CIS*	3.507	0.067	0.917	13.413	1.680	0.553	0.303	9.308
**Tumor Grade**	*Low (R)* vs. *high grade*	4.783	**<0.001**	2.947	7.764	1.933	**0.030**	1.065	3.511
**Single Dose IVC**	*Yes (R)* vs. *no*	9.442	<**0.001**	3.326	26.805	3.642	**0.025**	1.178	11.266
**Intravesical Treatment**	*No*		<**0.001**				**0.003**		
	*Chemotherapy*	0.439	**<0.001**	0.301	0.641	0.279	**0.015**	0.100	0.777
	*BCG*	0.591	**0.002**	0.426	0.819	0.427	**0.006**	0.233	0.785

OR: odds ratio; CI: confidence interval; R: Reference category; ASA: American Society of Anesthesiologists; M. propria: Muscularis propria; CIS: carcinoma in situ; IVC: intravesical chemotherapy; BCG: or Bacillus Calmette–Guérin; variables with *p* < 0.05 in univariable analysis were entered into the multivariable model.

**Table 3:** Univariable and multivariable Cox regression analysis for predictors of PFS.

Factor		Univariable Analysis				Multivariable Analysis			
		HR	*p*-Value	%95 CI		HR	*p*-Value	%95 CI	
				Lower	Upper			Lower	Upper
**Age**	*Years*	1.030	**0.001**	1.012	1.048	1.010	0.364	0.988	1.032
**Gender**	*Male (R)* vs. *female*	1.016	0.953	0.590	1.752				
**ASA**	1		0.152				0.472		
	2	4.231	0.072	1.015	17.629	2.561	0.207	0.595	11.023
	3	5.021	**0.046**	1.211	20.818	2.547	0.221	0.569	11.389
	4	6.979	**0.028**	1.489	32.705	3.595	0.123	0.706	18.306
**Presence of M. Propria**	*Yes (R)* vs. *no*	1.125	0.535	0.775	1.634				
**Tumor M** **ultiplicity**	*Number*	1.083	0.171	0.996	1.213				
**Tumor Size**	*mm*	1.013	**<0.001**	1.008	1.018	0.997	0.407	0.990	1.004
**Early Recurrence**	*No (R)* vs. *yes*	9.145	**<0.001**	6.038	13.849	6.053	**<0.001**	3.806	9.628
**Tumor Morphology**	*Papillary (R)*		**<0.001**				0.188		
	*Solid*	2.542	**<0.001**	1.734	3.726	1.497	0.068	0.971	2.310
	*Flat*	1.160	0.883	0.161	8.380	0.945	0.963	0.087	10.208
**Tumor Pathology**	*Ta (R)*		**<0.001**				0.110		
	*T*1	3.798	**<0.001**	2.426	5.947	1.795	**0.038**	1.032	3.124
	*Concomitant CIS*	2.246	0.186	0.677	7.456	1.287	0.739	0.291	5.698
**Tumor Grade**	*Low (R)* vs. *high grade*	3.373	**<0.001**	2.282	4.984	1.518	0.084	0.945	2.438
**Single Dose IVC**	*Yes (R)* vs. *no*	3.167	**0.001**	1.650	6.079	0.952	0.898	0.450	2.015
**Intravesical Treatment**	*No*		**<0.001**				**0.019**		
	*Chemotherapy*	0.250	**<0.001**	0.132	0.473	0.565	0.122	0.274	1.165
	*BCG*	0.538	**0.015**	0.377	0.901	0.532	**0.009**	0.332	0.854

HR: hazard ratio; CI: confidence interval; R: Reference category; ASA: American Society of Anesthesiologists; M. propria: Muscularis propria; CIS: carcinoma in situ; IVC: intravesical chemotherapy; BCG: or Bacillus Calmette–Guérin; variables with *p* < 0.05 in univariable analysis were entered into the multivariable model.

**Table 4:** Univariable and multivariable Cox regression analysis for predictors of OS.

Factor		Univariable Analysis				Multivariable Analysis			
		HR	*p*-Value	%95 CI		HR	*p*-Value	%95 CI	
				Lower	Upper			Lower	Upper
**Age**	*Years*	1.051	**<0.001**	1.035	1.068	1.031	**0.001**	1.012	1.051
**Gender**	*Male (R)* vs. *female*	1.299	0.230	0.847	1.993				
**ASA**	1		**<0.001**				**0.008**		
	2	1.765	0.231	0.696	4.476	0.839	0.723	0.317	2.220
	3	3.694	**0.005**	1.495	9.129	1.378	0.532	0.504	3.768
	4	6.080	**<0.001**	2.216	16.680	2.524	0.109	0.814	7.830
**Presence of M. Propria**	*Yes (R)* vs. *no*	1.015	0.926	0.738	1.397				
**Tumor M** **ultiplicity**	*Number*	1.087	**0.035**	1.006	1.176	0.999	0.979	0.896	1.113
**Tumor Size**	*mm*	1.012	**<0.001**	1.008	1.016	1.003	0.289	0.997	1.009
**Early Recurrence**	*No (R)* vs. *yes*	3.463	**<0.001**	2.520	4.759	1.961	**<0.001**	1.351	2.848
**Tumor Morphology**	*Papillary (R)*		**<0.001**				**0.026**		
	*Solid*	2.663	**<0.001**	1.911	3.711	1.664	**0.007**	1.151	2.406
	*Flat*	0.000	0.962	0.000	>10^5^	0.000	0.952	0.000	>10^5^
**Tumor Pathology**	*Ta (R)*		**<0.001**				0.068		
	*T*1	2.990	**<0.001**	2.097	4.262	1.679	**0.024**	1.069	2.636
	*Concominant CIS*	0.887	0.869	0.214	3.671	1.027	0.972	0.237	4.449
**Tumor Grade**	*Low (R)* vs. *high grade*	2.900	**<0.001**	2.103	3.998	1.387	0.101	0.938	2.052
**Single Dose IVC**	*Yes (R)* vs. *no*	2.324	**<0.001**	1.462	3.696	1.147	0.620	0.667	1.971
**Intravesical Treatment**	*No*		**<0.001**				0.472		
	*Chemotherapy*	0.389	**<0.001**	0.244	0.619	0.801	0.425	0.464	1.382
	*BCG*	0.700	0.060	0.483	1.015	0.803	0.280	0.538	1.196

HR: hazard ratio; CI: confidence interval; R: Reference category; ASA: American Society of Anesthesiologists; M. propria: Muscularis propria; CIS: carcinoma in situ; IVC: intravesical chemotherapy; BCG: or Bacillus Calmette–Guérin; variables with *p* < 0.05 in univariable analysis were entered into the multivariate model.

## Data Availability

The datasets used and/or analyzed during the current study are available from the corresponding author on reasonable request.

## Supplementary Materials

The following supporting information can be downloaded at: https://www.mdpi.com/article/10.3390/jcm15062463/s1, Table S1: Sensitivity analysis using conventional progression definition (≥T2); Table S2: Univariable and multivariable Cox regression analysis for predictors of CSS.
