# Supplementary material for: Early Recurrence Following Complete Initial Resection Predicts Adverse Oncological Outcomes in NMIBC
Source: J Clin Med. 2026 Mar 23;15(6):2463. doi: 10.3390/jcm15062463 (PMC13027191; doi:10.3390/jcm15062463)
Supplement: Supplementary file 1 [file jcm-15-02463-s001.zip › jcm-4174815-supplementary.pdf]

Supplementary Table S1: Sensitivity analysis using conventional progression definition (≥T2).

Factor		Univariate Analysis				Multivariate Analysis			
				%95 CI				%95 CI	
		HR	p-value	Lower	Upper	HR	p-value	Lower	Upper
**Age**	*Years*	1.027	**0.016**	1.005	1.050	1.007	0.587	0.983	1.030
**Gender**	*Male (R) vs female*	1.086	0.809	0.556	2.122				
**ASA**	*1*		0.356						
	2	2.317	0.254	0.547	9.821				
	3	2.696	0.174	0.645	11.270				
	4	3.955	0.093	0.794	19.703				
**Presence of M.Propria**	*Yes (R) vs no*	0.955	0.851	0.593	1.538				
**Tumor Multiplicity**	*Number*	1.148	**0.040**	1.006	1.311	1.145	0.103	0.973	1.347
**Tumor Size**	*mm*	1.011	**0.001**	1.005	1.018	0.992	0.084	0.984	1.001
**Early Recurrence**	*No (R) vs yes*	6.915	**<0.001**	4.187	11.420	3.909	**<0.001**	2.234	6.839
**Tumor Morphology**	*Papillary (R)*		**<0.001**				0.309		
	Solid	2.704	**<0.001**	1.667	4.385	1.517	0.125	0.890	2.587
	Flat	0	0.973	0	$>10^5$	0.001	0.986	0	
**Tumor Pathology**	*Ta (R)*		**<0.001**				0.082		
	T1	5.326	**<0.001**	2.855	9.935	2.337	**0.025**	1.111	4.916
	Concomitant CIS	0	0.890	0	$>10^5$	0	0.970	0	$>10^5$
**Tumor Grade**	*Low (R) vs high grade*	5.728	**<0.001**	3.298	9.950	2.846	**0.002**	1.448	5.594
**Single Dose IVC**	*Yes (R) vs no*	3.231	**0.006**	1.394	7.493	0.796	0.642	0.304	2.085
**Intravesical Treatment**	*No*		**0.002**				0.126		
	Chemotherapy	0.237	**0.001**	0.101	0.556	0.684	0.444	0.259	1.807
	BCG	0.629	0.097	0.365	1.087	0.553	**0.047**	0.308	0.992

HR: hazard ratio; CI: confidence interval; R: Reference category; ASA: American Society of Anesthesiologists; M.propria: Muscularis propria; CIS: carcinoma in situ; IVC: intravesical chemotherapy; BCG: or Bacillus Calmette-Guérin;

Variables with p < 0.05 in univariate analysis were entered into the multivariate model.

Supplementary Table S2: Univariate and multivariate Cox regression analysis for predictors of CSS.

**Factor**		**Univariate Analysis**				**Multivariate Analysis**			
				%95 CI				%95 CI	
		HR	p-value	Lower	Upper	HR	p-value	Lower	Upper
**Age**	*Years*	1.049	**<0.001**	1.026	1.074	1.026	0.062	0.999	1.055
**Gender**	*Male (R) vs. Female*	1.317	0.383	0.709	2.445				
**ASA**	*1*		**0.009**				0.298		
	2	3.788	0.194	0.509	28.216	1.720	0.603	0.222	13.291
	3	7.675	**0.044**	1.055	55.848	2.690	0.351	0.336	21.558
	4	9.609	**0.037**	1.152	80.123	3.952	0.229	0.420	37.149
**Presence of M.Propria**	*Yes (R) vs. No*	1.236	0.365	0.782	1.956				
**Tumor Multiplicity**	*Number*	1.067	0.295	0.945	1.205				
**Tumor Size**	*mm*	1.014	**<0.001**	1.008	1.019	1.002	0.559	0.995	1.010
**Early Recurrence**	*No (R) vs. Yes*	4.695	**<0.001**	2.921	7.546	2.052	**0.009**	1.196	3.520
**Tumor Morphology**	*Papillary (R)*		**<0.001**				**0.021**		
	Solid	3.748	**<0.001**	2.349	5.980	2.058	**0.005**	1.239	3.419

	Flat	0.000	0.963	0.000	>10^5	0.000	0.967	0.000	>10^5
**Tumor Pathology**	*Ta (R)*		**<0.001**				**0.017**		
	T1	6.507	**<0.001**	3.333	12.704	3.143	**0.005**	1.425	6.934
	Concominant CIS	3.471	0.109	0.759	15.876	3.466	1.321	0.689	17.441
**Tumor Grade**	*Low (R) vs. high grade*	4.097	**<0.001**	2.482	6.765	1.355	0.304	0.759	2.420
**Single Dose IVC**	*Yes (R) vs. No*	4.416	**0.001**	1.775	10.984	1.480	0.447	0.539	4.067
**Intravesical Treatment**	*No*		**0.001**				0.482		
	Chemotherapy	0.220	**<0.001**	0.094	0.514	0.749	0.556	0.286	1.963
	BCG	0.621	**0.084**	0.362	1.066	0.709	0.250	0.395	1.273
